# Differences in Cadmium Uptake and Accumulation in Seedlings of Wheat Varieties with Low- and High-Grain Cadmium Accumulation under Different Drought Stresses

**DOI:** 10.3390/plants12193499

**Published:** 2023-10-08

**Authors:** Yatao Xiao, Wei Guo, Xuebin Qi, Mahmoud S. Hashem, Dezhe Wang, Chaoxiang Sun

**Affiliations:** 1 Institute of Farmland Irrigation of CAAS/Key Laboratory of High-Efficient and Safe Utilization of Agriculture Water Resources, Chinese Academy of Agricultural Sciences, Xinxiang 453003, China; xiaoyatao@henau.edu.cn; 2 College of Mechanical and Electrical Engineering, Henan Agricultural University, Zhengzhou 450002, China; wangzhe823@126.com (D.W.);; 3 Agricultural Research Center, Agricultural Engineering Research Institute (AEnRI), Giza 256, Egypt

**Keywords:** drought stress, cadmium, uptake and accumulation, wheat varieties, low- and high-grain Cd accumulation

## Abstract

Cadmium (Cd) and drought, as abiotic stresses, have long been significant challenges for crop growth and agricultural production. However, there have been relatively few studies conducted on the effects of drought stress on Cd uptake, especially regarding the differences in Cd uptake characterization in varieties with varying Cd accumulation under different drought stress. To investigate the effects of drought conditions on Cd uptake by wheat in different genotypes under specific background levels of Cd pollution, we validated the differences in root absorption characteristics of low- (YM) and high-grain Cd accumulating wheat genotypes (XM) using non-invasive micro-test technology, and we conducted a hydroponic experiment on the Cd addition and different drought levels in a climate-controlled chamber. The biomass, root morphology, Cd uptake, and accumulation were determined under Cd (100 µmol L^−1^) and different drought levels of 0% (0 MPa), 5% (−0.100 Mpa), 10% (−0.200 Mpa), and 15% (−0.388 Mpa) simulated by polyethylene glycol (PEG-6000). We found that the simultaneous exposure to Cd and drought had a suppressive effect on the total root lengths, root surface areas, and root volumes of XM and YM, albeit with distinct patterns of variation. As the concentration of PEG-6000 increased, the Cd concentrations and the amount of Cd accumulated in the roots and shoots of XM and YM decreased. Specifically, the Cd concentration in the roots exhibited a reduction ranging from 12.51% to 66.90%, while the Cd concentration in the shoots experienced an even greater decrease of 50.46% to 80.57%. The PEG-6000 concentration was significantly negatively correlated (*p* < 0.001) with Cd concentration of roots and shoots and Cd accumulation in roots, shoots, and the whole plants and significantly negatively correlated (*p* < 0.05) with the total length, surface area, and volume of roots. This study confirms that drought stress (5% PEG-6000) can decrease the uptake and accumulation of Cd in wheat seedlings without significant inhibition of biomass, and the change of root morphology (root length) and the decrease of Cd concentration in roots may be the main direct pathways for achieving these effects under drought stress. This research provides a new perspective and idea for water management in Cd-contaminated farmland.

## 1. Introduction

Cadmium (Cd) is a highly toxic heavy metal that is widely used and known to cause serious soil pollution [1,2]. In the ecosystem, Cd present in the soil can be taken up by plants and participates in various biochemical processes within organisms, which can disrupt normal cell metabolism, cause DNA damage, impair the DNA repair system, and ultimately result in cell apoptosis [3,4,5]. The excessive accumulation of Cd directly impedes plant growth and development, reduces crop yield, and gives rise to crop quality and safety concerns [6,7]. Moreover, Cd can biomagnify in the food chain, posing a threat to human health [8]. Wheat (*Triticum aestivum* L.), one of the most important grain crops in the world, has garnered considerable attention from many scholars due to the excessive accumulation of Cd in its seeds [9]. It has been shown that the consumption of processed Cd-contaminated wheat seeds is a significant pathway for Cd intake in humans [10,11]. Therefore, ensuring the quality of wheat is of utmost importance for public health and safety.

The process of global industrialization has led to the emergence of environmental concerns in many countries, particularly with regard to elevated levels of Cd in soil [12,13]. In response, various soil remediation technologies have been proposed, both domestically and internationally, including the chemical leaching method [14], electrochemical method [15], microbiological method [16], and phytoremediation [17]. However, the widespread adoption and implementation of these existing soil remediation technologies face challenges due to factors such as high treatment costs, lengthy remediation periods, and significant ecological consequences. Modifying farming management systems to align with local conditions, thereby diminishing the bioeffectiveness of Cd, curtailing the transfer of Cd from soil to crops, and reducing the accumulation of Cd in cereals [18], represents a more comprehensive and considerate approach to management.

Irrigation is an essential aspect of agricultural farming management. According to global statistics, a total of 3.34 × 10^8^ hm^2^ of cultivated land worldwide utilizes irrigation equipment for agricultural production, of which the largest irrigated area of the countries is China (7.3 × 10^7^ hm^2^), India (7 × 10^7^ hm^2^), and the United States (2.7 × 10^7^ hm^2^) [19]. The primary objective of irrigation is to regulate soil moisture levels, as insufficient moisture can lead to drought stress. Severe drought stress causes the closure of plant leaf stomata [20], resulting in a reduction in transpiration rate and leaf net photosynthetic rate [21]. This phenomenon disrupts chlorophyll synthesis [22], impedes crop growth and development, and alters both root and surface morphology [23]. Nevertheless, research has demonstrated that implementing appropriate deficit irrigation techniques can effectively enhance the physiological characteristics of the root system and plants [24,25]. This, in turn, improves photosynthetic efficiency and strengthens the oxidase defense system, ultimately positively influencing the quality and nutritional value of crops [26]. Studies indicate that drought stress induces changes in root morphology and affects the uptake of Cd in plants [27]. According to Peng et al. [21], drought stress can diminish the capacity of plants to absorb Cd, and the extent of reduction is influenced by the severity of stress and the growth stage of the plants. Furthermore, it has been proposed that drought stress triggers oxidative stress, leading to a decrease in antioxidant enzyme activities and an increase in Cd concentration in plants [28]. The variability in their findings can be attributed to factors such as crop cultivars, drought levels, and crop developmental stages across relevant research studies. Furthermore, there are notable differences in the uptake and accumulation traits of Cd among different cultivars of the same crop.

Therefore, in this study, non-invasive micro-test technology (NMT) was used to verify the difference in Cd uptake by roots of wheat varieties with low- and high-Cd grain accumulation at the seedling stage. Subsequently, a drought simulation experiment utilizing polyethylene glycol (PEG-6000) was conducted to investigate the uptake characteristics of Cd and the morphological alterations in wheat seedlings under varying levels of drought stress, as well as the differences between two wheat cultivation varieties (low- and high-grain Cd-accumulating genotype). This study aims to provide a theoretical basis and research ideas for water management of farmland contaminated with Cd.

## 2. Results

### 2.1. Cd Absorption Characteristics

**Figure 1:**
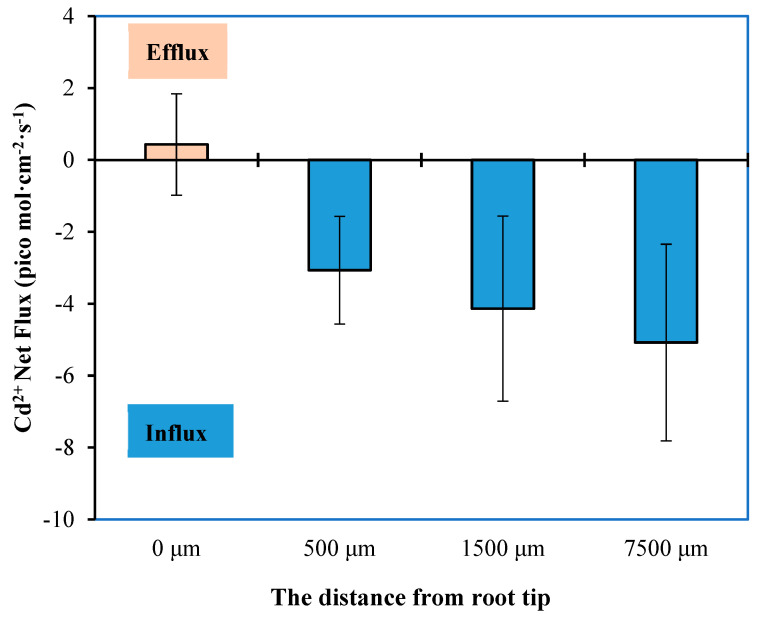
The mean Cd flux rates in different regions of XM roots at 100 µmol L^−1^ Cadmium (Cd) stress (pH = 6.0).

The roots, being the primary organ responsible for water and mineral absorption, exhibit varying rates of Cd absorption when exposed to a Cd environment. Figure 1 illustrates the occurrence of Cd efflux at the measurement point (0, 500, 1500, and 7500 µm) located at the root tip. As the distance between the measurement point and the root tip progressively increased, the real-time Cd influx rate exhibited a corresponding increase. Ultimately, the maximum inflow rate of Cd by root cells was determined at the measurement point situated 7500 µm away from the root tip, measuring 5.078 pico mol cm^−2^ s^−1^.

**Figure 2:**
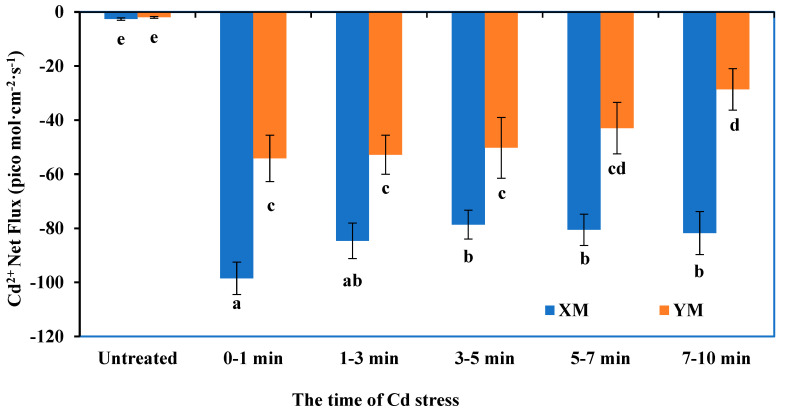
Genotypic variation of mean Cd flux rate at 7500 µm from the root tip of XM and YM under 100 µmol L^−1^ Cadmium (Cd) stress. The different letters indicate significant differences at *p* < 0.05 according to a least significant difference (LSD) test.

According to the results of the Cd flux rate in different regions of XM roots (as shown in Figure 1), we chose to measure and compare the Cd flux rate of XM and YM seedling roots at a distance of 7500 um from the root tip. The results, depicted in Figure 2, indicate that after adding 5 mL of measuring solution (consisting of 1 µmol L^−1^ CdCl_2_, 1 µmol L^−1^ CaCl_2_, and pH 6.0), there was a small Cd influx observed in both XM (−2.606 pico mol cm^−2^ s^−1^) and YM (−1.948 pico mol cm^−2^ s^−1^) roots. However, upon the addition of 100 µmol L^−1^ CdCl_2_, the real-time flow rates of Cd at a distance of 7500 µm from the root tip of XM and YM roots were found to be highest within 1 min, reaching 98.484 pico mol cm^−2^ s^−1^ and 54.114 pico mol cm^−2^ s^−1^, respectively. During the subsequent measurement period, the real-time influx rates of Cd by the YM wheat varieties exhibited a consistent downward trend (2.446–47.124%). XM showed a trend of firstly decreasing and then slightly increasing, and the change range was 4.081% to 20.184%. Upon comparing the influx rates of Cd by the roots of XM and YM at this specific point (7500 µm), it was observed that XM exhibited a significantly higher influx rate than YM (*p* < 0.05). As the testing duration increased, the difference in the real-time influx rate between XM and YM reached a maximum of −53.115 pico mol cm^−2^ s^−1^ within the 7–10 min timeframe. Hence, it can be concluded that XM and YM, as two distinct wheat varieties, display notable disparities in their uptake of Cd.

### 2.2. Biomass and Root–Shoot Ratio

As shown in Table 1, compared with the Cd-only treatment (i.e., 0% PEG-6000), the PEG-6000 treatments had no significant effect on the dry biomass of roots (*p* > 0.05), either in the XM or YM. The shoots biomass of XM and YM tended to decrease with increasing concentration of the PEG-6000, and the differences reached significant levels at the 10% and 15% PEG-6000 treatments compared to the Cd-only treatment for shoots (*p* < 0.05). As the concentration of PEG-6000 increased, the whole plant biomass of XM and YM gradually decreased, with XM experiencing a maximum reduction of 46.99% and YM experiencing a maximum reduction of 36.43%. Meanwhile, the root–shoot ratios showed increasing trends, ranging from 19.71% to 137.96% in XM and from 20.74% to 99.63% in YM. The root–shoot ratios of XM in the 0%, 5%, 10%, and 15% PEG-6000 treatments were 1.02, 1.01, 1.11, and 1.21 times higher than those of YM, respectively; however, only the difference in the 15% PEG-6000 treatment was significant (*p* < 0.05). The shoots dry biomass, whole plant dry biomass, and root–shoot ratio were significantly influenced by the drought stress (*p* < 0.001).

### 2.3. Cd Concentration and Accumulation

As shown in Table 2, when the concentration of PEG-6000 increased, the Cd concentration in the roots and shoots of XM and YM significantly decreased (by 22.60–62.84% in XM roots, 12.51–66.9% in YM roots; 50.46–78.7% in XM shoots, 56.87–80.57% in YM shoots), and the differences between the different treatments were all significant (*p* < 0.05). The effects of drought stress induced by PEG-6000 on reducing Cd concentration in shoots (by 50.46~80.57%) were greater than the effect on roots (by 12.51~66.90%). When comparing the two wheat varieties, the Cd concentrations in the roots and shoots of YM were higher than that of XM in the Cd-only treatment, and the difference in Cd concentration in shoots reached a significant level (*p* < 0.05). In the 5% PEG-6000 treatment, the Cd concentrations in the roots of YM were significantly higher (1.16 times) than those in XM (*p* < 0.05). In the 10% PEG-6000 treatment, the Cd concentrations in the roots and shoots of YM were significantly higher (*p* < 0.05; 1.13 and 1.56 times, respectively) than those of XM. However, in the 15% PEG-6000 treatment, there was no significant difference in the Cd concentration of roots and shoots between XM and YM (*p* > 0.05).

The translocation factor (TF) is often used to reflect the plant’s ability to transport Cd upward. As shown in Table 2, the TFs of XM and YM under PEG-6000 treatments were lower than that of the Cd-only treatment (Cd-only), and the TFs of XM and YM showed the same changing trend with the increase of PEG-6000 concentration. The TF of XM reached a minimum of 0.297 in the 15% PEG-6000 treatment, while the TF of YM reached a minimum of 0.280 in the 5% PEG-6000 treatment. Comparing the two wheat varieties, the TFs of YM in the Cd-only, 10%, and 15% PEG-6000 treatments increased by 9.44%, 38.54%, and 12.46%, respectively, compared to XM, whereas the TF of YM decreased by 15.66% in the 5% PEG-6000 treatment compared to XM. In conclusion, the root Cd concentration, shoot Cd concentration, and TFs were significantly influenced by the varieties, drought stress, and their interaction (*p* < 0.001).

Table 3 revealed that Cd accumulation in the roots and shoots decreased significantly with the increase of drought stress induced by PEG-6000. The maximum reduction was 63.12% in XM roots, 65.12% in YM roots, 91.33% in XM shoots, and 87.09% in YM shoots. The amounts of Cd accumulated in the whole plants of YM significantly decreased with the increase of the PEG-6000 concentration (*p* < 0.05). The highest amount of Cd accumulated in the roots of YM was in the 5% PEG-6000 treatment (11.136 µg), which was not significantly different from that of the Cd-only treatment (*p* > 0.05) but was significantly higher than those of the other treatments (*p* < 0.05). Comparing the two wheat varieties, in the 5% PEG-6000 treatment, the amounts of Cd accumulated in the roots, shoots, and whole plants of YM were 1.24, 1.05, and 1.14 times higher than those of XM, respectively, but with no significant differences (*p* > 0.05). In the 10% and 15% PEG-6000 treatments, the amounts of Cd accumulated in the roots of XM and YM were not significantly different (*p* > 0.05) but were significantly lower (*p* < 0.05) than those of the Cd-only and 5% PEG-6000 treatments. The Cd accumulation in the root, shoot, and whole plants was significantly influenced by the drought stress induced by PEG-6000 treatments (*p* < 0.001). Otherwise, the Cd accumulation in the roots was significantly influenced by the interaction of varieties and drought stress (*p* < 0.05).

### 2.4. Root Morphology

**Figure 3:**
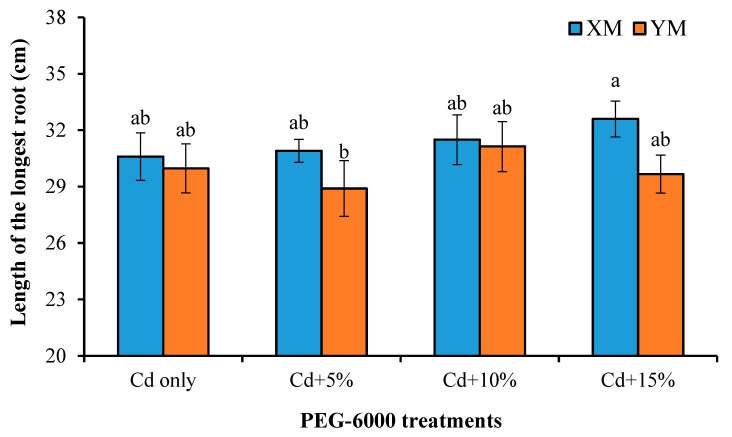
Effects of different drought stress induced by polyethylene glycol (PEG-6000) treatments on the longest root lengths of XM (high-grain Cadmium (Cd)-accumulating genotype) and YM (low-grain Cd-accumulating genotype) under 100 µmol L^−1^ of Cd stress. The different letters indicate significant differences at *p* < 0.05 according to a least significant difference (LSD) test.

Drought stress can stimulate the downward growth of roots, allowing them to access sufficient water and nutrients from deeper soil layers. The root length can reflect the vertical distribution of crop roots and drought resistance and represents the potential of root morphological changes to avoid polluted topsoil to some extent. As shown in Figure 3, compared with the Cd-only treatment, the longest root length of XM increased (0.98–6.54%) after adding drought stress, and it was proportional to the concentration of PEG-6000 treatment, while the longest root length of YM was reduced by 3.57% in the 5% PEG-6000 treatment, and then the maximum length of 31.13 cm was obtained in the 10% PEG-6000 treatment. Comparing XM and YM, the longest root length of XM was greater than YM under each treatment. However, according to the results of statistical analysis, the differences between XM and YM in each treatment and among varieties did not reach a significant level (*p* > 0.05). Due to the fact that root length extension will not obtain water supplement, the changes and differences of the longest roots of XM and YM can only be used as the characteristics of root change trend and cannot be used as a basis for judging root morphology construction.

**Figure 4:**
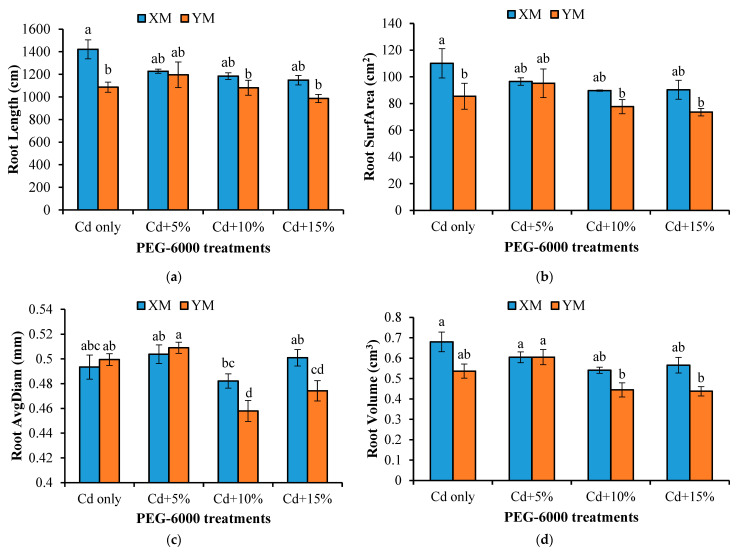
Effects of different drought stress induced by polyethylene glycol (PEG-6000) treatments on the root lengths (**a**), root surface areas (“Root SurfArea”) (**b**), average root diameters (“Root AvgDiam”) (**c**), and root volumes (**d**) of XM (high-grain Cadmium (Cd)-accumulating genotype) and YM (low-grain Cd-accumulating genotype) under 100 µmol L^−1^ of Cd stress. The different letters indicate significant differences at *p* < 0.05 according to a least significant difference (LSD) test.

Figure 4 shows that the lengths, surface areas, diameters, and volumes of roots were affected by the PEG-induced drought stress. Compared to the Cd-only treatment, the length, surface area, and volume of XM roots under the 5% PEG-6000 treatment were reduced by 13.66%, 12.38%, and 11.08%, respectively. With the increase of the PEG-6000 concentration, the root length of XM showed a gradual decreasing trend; however, there was no significant difference between treatments (*p* > 0.05). The root surface area and root volume of XM also gradually decreased as the PEG-6000 concentration increased from 0% to 10% but then increased in the 15% PEG-6000 treatment. In the 5%, 10%, and 15% PEG-6000 treatments, the root diameters of XM were 1.02, 0.98, and 1.02 times larger than those of the Cd-only treatment, respectively. Comparatively, the length, surface area, and volume of YM roots increased by 10.07%, 11.35%, and 12.87% in the 5% PEG-6000 treatment, respectively, when compared to the Cd-only treatment. However, these measurements decreased with further increases in the PEG-6000 concentration, and no significant differences were observed between the treatments (*p* > 0.05). The 5% PEG-6000 treatment led to an increase in the root diameter of YM compared to the Cd-only treatment (*p* > 0.05). However, when the PEG-6000 concentration increased to 10% and 15%, the root diameters of YM were significantly lower than those in the Cd-only and 5% PEG-6000 treatments (*p* < 0.05). When comparing XM and YM under the drought stress treatments, the length, surface area, and volume of roots in XM were all larger than those of YM, but the differences were not significant (*p* > 0.05). The root diameters of XM in the 10% and 15% PEG-6000 treatments were 1.04 and 1.06 times larger than those of YM, respectively, with significant differences observed in the 10% and 15% PEG-6000 treatments (*p* < 0.05).

To further analyze the effects of the PEG-induced drought stress on the root morphologies of XM and YM, the roots were categorized into fine roots (0–1 mm) and thick roots (>1 mm). As indicated in Table 4, the root characteristics of both fine and thick roots of XM and YM exhibited significant changes with different PEG-6000 concentrations. For the fine roots of XM, the length, surface area, and volume under the 5% PEG-6000 treatment were 0.86, 1.00, and 1.05 times larger than those of the Cd-only treatment, respectively, with only the root length showing a significant difference (*p* < 0.05). For the thick roots of XM, the surface area and volume were significantly higher in the 5% PEG-6000 treatment compared to the other treatments (*p* < 0.05). When increasing PEG-6000 concentration, the surface areas of thick roots initially increased, then decreased, and ultimately increased again.

For the fine roots of YM, the length, surface area, and volume in the 5% PEG-6000 treatment increased by 9.99%, 12.42%, and 31.10%, respectively, compared to the Cd-only treatment. The root length and volume showed significant differences between these two treatments (*p* < 0.05). In the 5% PEG-6000 treatment of YM, the indices of thick roots were significantly higher than those of the Cd-only treatment (*p* < 0.05). However, the lengths, surface areas, and volumes of fine and thick roots decreased after the 5%, 10%, and 15% PEG-6000 treatments of YM, and the differences in the root indices of fine and thick roots between treatments were significant (*p* < 0.05).

The differences in the morphological parameters of roots between XM and YM were significant (*p* < 0.05). In the Cd-only treatment, the root length, surface area, and volumes of XM were significantly higher than those of YM (*p* < 0.05). In the 5% PEG-6000 treatment, the fine root length, surface area, and volume of YM were 0.97, 0.99, and 1.07 times larger than those of XM, respectively, but the differences were not significant (*p* > 0.05). In contrast, the thick root length, surface area, and volume of YM were 0.73, 0.80, and 0.83 times than those of XM, respectively, and the differences were significant (*p* < 0.05). In the 10% PEG-6000 treatment, the think and fine root parameters of YM were slightly lower than those of XM, but only the differences in the fine root volume and length between XM and YM were significant (*p* < 0.05). When exposed to 15% PEG-6000, the root length and root volume of YM were significantly lower than those of XM (*p* < 0.05). The root length, surface area, and volume of thick roots were significantly influenced by the varieties, stress level, and their interaction (*p* <0.001). The root surface area of fine roots was significantly influenced by the varieties (*p* < 0.05).

### 2.5. Correlation Analysis

**Figure 5:**
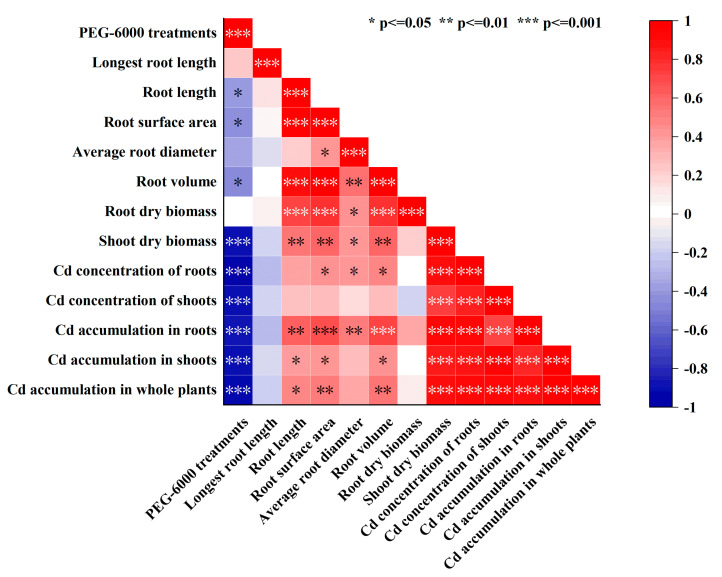
Correlation coefficient analysis of polyethylene glycol (PEG-6000) treatments and morpho-physiological traits. * Correlation significant at the 0.05 level (2-tailed). ** Correlation significant at the 0.01 level (2-tailed). *** Correlation significant at the 0.001 level (2-tailed).

A correlation analysis was performed to examine the associations between PEG-induced drought stress and the root morphologies, as well as the concentration and accumulation of Cd in XM and YM. The findings (Figure 5) indicated highly significant negative correlations (*p* < 0.001) between PEG-6000 concentrations and shoot dry biomass, Cd concentration in roots and shoots, and Cd accumulation in roots, shoots, and entire plants. Significant negative correlations (*p* < 0.05) were observed between PEG-6000 concentrations and the length, surface area, and volume of roots. The Cd concentration in roots exhibited a highly positive correlation with the surface area, diameter, and volume of roots (*p* < 0.05; correlation coefficients of 0.456, 0.439, and 0.486, respectively), while the Cd concentration in shoots displayed a positive correlation with the Cd concentration in roots (*p* < 0.001; correlation coefficients of 0.860). The accumulation of Cd in the whole plant demonstrated a highly significant correlation with the dry biomass of shoots, Cd concentration in roots, Cd concentration in shoots, Cd accumulation in roots, and Cd accumulation in shoots (*p* < 0.001; 0.919, 0.933, 0.927, 0.914, and 0.986, respectively). Furthermore, it exhibited significant correlations with root surface area and root volume (*p* < 0.01; 0.534 and 0.549, respectively), as well as a significant correlation with root length (*p* < 0.05; 0.487). The correlation analysis results confirmed that augmenting the concentration of PEG-6000 treatment yielded a reduction in Cd concentration of both the roots and shoots, thereby impeding the accumulation of Cd in these plant parts. Simultaneously, this treatment exerted a substantial inhibitory impact on root morphology and shoot dry biomass. Consequently, the inhibitory effect of drought stress on Cd uptake and accumulation was meaningful only when plant biomass did not decrease significantly.

### 2.6. Path Analysis

**Figure 6:**
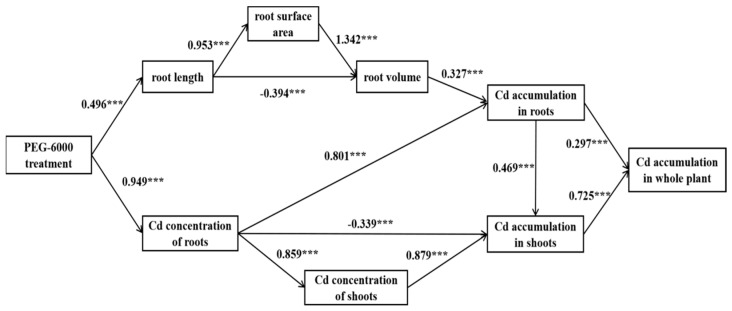
Path analysis of the relation between polyethylene glycol (PEG-6000) treatments and Cd accumulation in whole plant (*p* = 0.333, x^2^/df = 1.101, GFI = 0.952, RMSEA = 0.066, CFI = 0.995). *** significant at the 0.01 level.

Furthermore, we constructed a path relationship model (as shown in Figure 6) using root length, root surface area, root volume, Cd concentration in roots, Cd concentration in shoots, Cd accumulation in roots, and Cd accumulation in shoots that all had been confirmed to be significantly correlated with the Cd accumulation in the whole plant in the Pearson correlation coefficient analysis. The corresponding osmotic potentials of 0 MPa, −0.100 MPa, −0.200 MPa, and −0.388 MPa for each PEG-6000 treatment were input into the model, and the path analysis was carried out. The results showed that the impact of PEG-6000 treatments on root morphology was primarily manifested through alterations in root length, which subsequently affected the surface area of the roots and co-contributed to the overall development of root volume. The concentration of Cd in roots exhibited a noteworthy positive effect on the accumulation of Cd in roots and the concentration of Cd in shoots while displaying a significant negative effect on the accumulation of Cd in shoots. Additionally, the root volume also had a positive influence on the accumulation of Cd in the roots, while the accumulation of Cd in the shoots was positively influenced not only by the concentration of Cd in the shoots but also by the accumulation of Cd in the roots. Both the accumulation of Cd in the roots and shoots had positive effects on the Cd accumulation in the whole plant, with the effect of Cd accumulation in the shoots being greater than that in the roots.

## 3. Discussion

In this study, we verified the differential characteristics of Cd uptake by seedling roots of varieties with low- (XM) and high-grain Cd accumulation (YM) using NMT and conducted an analysis of the morphological changes and characteristics of Cd uptake and accumulation in wheat seedlings subjected to combined stresses of Cd (100 µmol L^−1^) and varying drought gradients (simulated by PEG-6000). Our findings revealed that the introduction of drought stress had a significant impact on biomass, root morphology, and Cd uptake and accumulation in wheat plants under Cd stress, and these effects varied among different varieties. These results were further supported by various related studies.

The process of plant growth and development is a prolonged adaptive mechanism that entails the continual improvement of the morphological structure and physiological function of plant organs. The growth and development of the root system, which serves as a crucial tissue for nutrient assimilation in plants, plays a decisive role in the plant’s capacity to absorb and transport water and nutrients [29]. In response to abiotic stress conditions, including drought, the root undergoes modifications to adapt to the challenging environment. Water availability is a crucial factor that stimulates root development [30,31]. Limited water supply can induce root morphogenesis [32] and promote deeper root distribution in the soil [33]. This enables plants to access water stored in deeper soil layers, ensuring their survival during drought periods [34]. However, excessive or prolonged drought stress adversely affects nutrient development in plant tissues and organs, impedes root morphological development, and disrupts the allocation of plant dry matter. This phenomenon is recognized as a significant mechanism by which plants adapt to drought conditions. Smucker et al. [35] conducted a study that revealed a positive correlation between decreased soil water content and increased assimilatory transport from aboveground areas to the root system. This leads to accelerated root growth as plants search for additional water resources. Consequently, root–crown ratio, total root length, and root surface area increase under decreased soil water content. Additionally, previous research has demonstrated that wheat plants can exhibit a root–crown ratio ranging from 40% to 80% under drought stress [36]. These findings highlight the significant role of soil water content in influencing plant root morphology and the accumulation and distribution of dry matter [37].

However, studying comprehensive root systems assessing root morphology, including vertical distribution, in traditional soil-based experiments can be challenging. In our investigation, we addressed this challenge by simulating various levels of drought stress using PEG-6000 under hydroponic conditions to obtain diverse intact root morphologies of the seedlings. The results indicated a decline in shoot biomass and overall plant biomass for both XM and YM, accompanied by an increase in the root–crown ratio as the concentration of PEG-6000 increased (Table 1). These outcomes are consistent with previous research findings. The results depicted in Figure 4 indicate a decrease in the total root length, root surface area, and root volume of XM as the concentration of PEG increased. Conversely, the total root length, root surface area, and root volume of YM initially increased under the 5% PEG treatment but subsequently decreased with higher PEG concentrations. These results suggest the existence of inter-varietal differences in the response of root morphology to drought stress. Notably, the total root length, root surface area, and root volume of YM reached their peak values under the 5% PEG treatment, supporting the viewpoint that appropriate drought stress can stimulate root development.

Root morphology is directly related to the uptake of water and mineral elements by crops [7,38]. Cd, as a non-essential element, can enter the plant through the apoplastic pathway and the symplastic pathway [39]. Therefore, the relationship between root morphology and Cd uptake has received much attention [40,41]. Zhang et al. [42] discovered that the characteristics of the wheat root system play a crucial role in Cd uptake and accumulation in plants. The primary source of Cd accumulation in wheat seeds is the direct uptake of Cd from the soil by the root system [41]. Cd accumulation in root tissues has been found to be positively correlated with root weight and crown root number [43]. Deng et al. [44] observed that the high accumulation of Cd in brown rice was linked to fine root diameter, large root surface area, long total root length, and a higher number of root tips. Similarly, Lu et al. [45] concluded that the accumulation of Cd in roots and fine roots exhibited a significant correlation with root morphology. Compared with thick roots, fine roots were more sensitive to Cd stress and played a crucial role in plant accumulation.

When it comes to coupled Cd and drought stress, it has been observed that drought stress reduces Cd uptake [46]. Under well-watered conditions, increased leaf transpiration promotes xylem flow from nutrient solution to the above-mentioned organs, enhancing the transport of the Cd process [47]. However, some studies have suggested that the decrease in Cd uptake during drought may be attributed to the inhibition of root growth and modification of root morphology rather than solely to a reduction in transpiration [48]. In our research, it was observed that the concentration of Cd in wheat plants decreased with increasing PEG-6000 concentration, indicating a significant correlation between root morphology (specifically, total length, surface area, and volume) and Cd accumulation. However, we believe that the process of Cd uptake and transportation from the roots to the aboveground parts of the plant is influenced by multiple factors. The application of PEG-6000 treatment induces an arid environment, which inevitably leads to a reduction in plant transpiration activity. This reduction hampers the absorption of Cd from the nutrient solution into the aboveground organs and the flow of Cd through the xylem, ultimately leading to a decrease in Cd translocation [47]. However, Cd uptake by roots is not directly dependent on or determined by transpiration. The reduction in total root length, surface area, and volume under the combined effect of drought and Cd stress may be one of the important factors influencing the decrease of Cd content within plants. The uptake and accumulation of Cd can be reduced by inhibiting the total length, surface area, and volume of roots [48]. Furthermore, root vitality is also a crucial factor in Cd uptake, and several studies have substantiated that higher root respiration intensity is responsible for the robust Cd accumulation capacity observed in plants [49].

While Cd uptake is influenced by multiple factors, the root system, as the main route for plants to absorb Cd from the soil, plays a crucial role in the uptake and accumulation of Cd. Liang et al. [50] pointed out that differences in root morphology characteristics among different spring wheat cultivars have been identified as important factors contributing to Cd accumulation in the kernels. This conclusion is supported by the differences in root morphology observed between low- and high-grain Cd-accumulating wheat varieties in the present study. In addition, we found that the PEG-6000 treatment increased the length of the longest root system (Figure 3), indicating that drought stress has the potential to modify the vertical distribution of the root system. This finding aligns with previous research demonstrating increased root biomass, surface area, and volume in the 20–40 cm soil layer of peanut varieties under drought conditions [51]. Considering that Cd contamination in soil predominantly occurs in the uppermost soil layer (0–20 cm, which had been confirmed in our previous research), we propose investigating the feasibility of inducing deeper root distribution as a means to mitigate Cd uptake by plants warrants further investigation.

In summary, we believe that the implementation of water management strategies in dryland farmland can yield substantial reductions in the levels of Cd found in plants. The alterations in root morphology and deep soil distribution driven by water will be one of the pivotal factors in reducing Cd concentration in plants and are effective for various genotypes of wheat cultivars. However, when employing water management measures like drought stress or deficit irrigation, it becomes imperative to carefully consider the implications for crop growth and development while also considering the ultimate goal of maximizing crop yield. Hence, the suitable drought stress gradients or water deficit levels, along with the implementation of timely and efficient water supply, will serve as crucial elements in the management of water resources in arid agricultural areas, necessitating further investigation and validation. Moreover, it is imperative to consider the impact of irrigation methods, such as underground drip irrigation, on the development and vertical distribution of root morphology.

## 4. Material and Methods

### 4.1. Plant Materials and Culturing

Trial site and seed varieties: The experiment was carried out in a climate-controlled chamber at the Agricultural Water and Soil Environment Field Scientific Observation and Experimental Station of the Chinese Academy of Agricultural Sciences. After a field screening trial, a high-grain Cd-accumulating genotype (Xinmai 9817 (XM)) and a low-grain Cd-accumulating genotype (Yaomai 16 (YM)) wheat were selected for this experiment.

**Figure 7:**
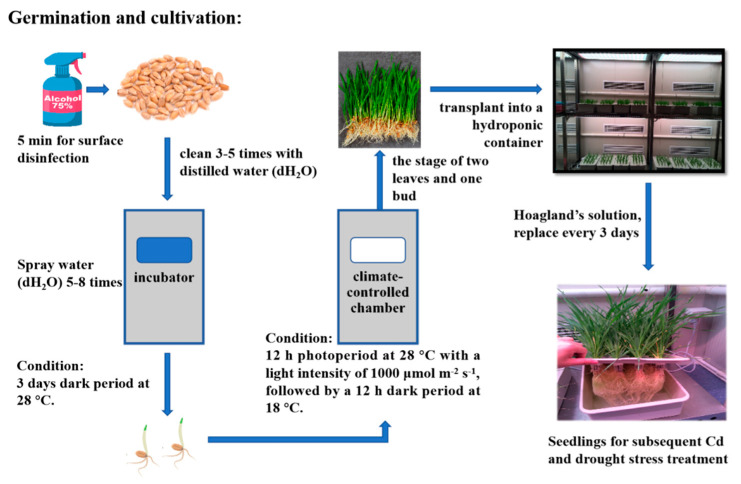
Schematic diagram of the germination and cultivation process.

Germination and cultivation: As shown in Figure 7, the seeds of XM and YM were soaked in 75% (*v/v*) ethanol for 5 min for surface disinfection and then rinsed thoroughly with distilled water (dH_2_O). The soaked seeds were placed on sterilized gauze and then moved to an incubator for germination at 28 °C. Distilled water was sprayed evenly on the surface of the seeds to moisten them. After complete emergence, seeds were transplanted onto breeding plates and cultivated in a climate-controlled chamber set to a 12 h photoperiod at 28 °C with a light intensity of 1000 μmol m^−2^ s^−1^, followed by a 12 h dark period at 18 °C. When the seeds were at the stage of two leaves and one bud, homogenous seedlings with their entire roots were transplanted into a hydroponic container and placed back in the climate-controlled chamber for further cultivation. The conditions for hydroponic experiments were strictly controlled, and the nutrient solution was replaced every three days. After sufficient plant biomass was obtained, the seedlings were moved to a particular nutrient solution for subsequent stress treatments.

### 4.2. Experiment on Measuring the Absorption Characteristics of Seedling Roots

Measurement of net Cd fluxes at different sites of roots: Using Non-invasive Micro-test (NMT) Technology Equipment (NMT Physiolyzer^®^, Younger USA LLC, Amherst; Xuyue (Beijing) Sci. & Tech. Co., Ltd.), we measured the real-time rate of Cd entering and exiting the wheat roots, which is the Cd flux. We chose pre-cultivated XM seedlings with favorable growth potential and subjected them to a 24 h preconditioning period in a 100 µmol L^−1^ CdCl_2_ solution. Subsequently, we utilized filter paper strips and resin blocks to secure the root of the sample at the base of the culture dish, thereby exposing the root tip region. We introduced the measuring solution (consisting of 100 µmol L^−1^ CdCl_2_, 100 µmol L^−1^ CaCl_2_, and pH 6.0) into the culture dish, submerged the roots, and allowed them to remain for 20 min. Then, we replaced the initial measuring solution, added 5 mL of fresh measuring solution, and proceeded to load the sample for testing. We located the test site of the wheat root tip under a microscope at distances of 0 μm, 500 μm, 1500 μm, and 7500 μm from the root tip. We positioned the Cd flux microsensor approximately 10 μm above the detection site on the root surface and commenced the testing process. Each site was subjected to a 3 min testing period, with each group undergoing 4 replicates. The Cd flow rate data was directly obtained using imFluxes V2.0 software (YoungerUSA LLC, Amherst, MA 01002, USA), with the flow rate unit expressed as pico mol cm^−2^ s^−1^.

Measurement of net Cd fluxes at a specific site of roots: Based on the findings pertaining to the net fluxes of Cd at various root locations of XM, the site (7500 μm away from the root tip) exhibiting the highest net absorption fluxes of Cd was designated as the NMT detection site for assessing the real-time rate of Cd entry and exit from roots. In accordance with the aforementioned methodology, XM and YM seedlings were carefully chosen and immobilized, and the relevant sections were subjected to leakage. Subsequently, a measuring solution (consisting of 1 µmol L^−1^ CdCl_2_, 1 µmol L^−1^ CaCl_2_, and pH 6.0) was introduced into a Petri dish to fully submerge the roots, allowing for a 20 min period of rest. The measuring solution was then discarded, and 5 mL of fresh measuring solution was added for subsequent analysis of the sample. During microscopic examination, a specific point located 7500 μm away from the root tip was identified, and the Cd flux microsensor was positioned approximately 10 μm above the designated area for detection. Following 5 min of data collection, a 100 µmol L^−1^ CdCl_2_ solution was introduced to the Petri dish, and data collection continued for an additional 10 min until no significant changes in the signal were observed. Each group underwent 8 replicates for data collection. The imFluxes V2.0 software (YoungerUSA LLC, Amherst, MA 01002, USA) was employed to directly read the Cd flux data.

### 4.3. Hydroponic Experiment of Drought Coupled with Cd Stress on Seedlings

Experimental treatment: The drought treatment was performed with PEG-6000. The PEG-6000 concentration gradient was set as 0% (control), 5%, 10%, and 15% (M/V) by adding PEG-6000 to Hoagland’s solution [48] with a Cd concentration of 100 µmol L^−1^ [52]. According to Michel et al. [53], the osmotic potential(cs) of 5%, 10%, and 15% PEG-6000 are approximately −0.100 MPa, −0.200 MPa, and −0.388 MPa, respectively. Each treatment was repeated three times, with a total of 24 experimental units carried out in the experiment. After 14 days of exposure to Cd and PEG-6000, the seedlings were harvested according to the root/shoot parts and washed well for subsequent analysis.

Determination of root parameters: The root system was scanned using a root scanner (EPSON V750) to obtain image information, and the WinRHIZO Pro 2008 analysis software was used to obtain related indices, such as the total length, surface area, average diameter, and volume of roots.

Determination of the root/shoot ratio: Five seedlings were harvested according to their roots and shoots and then placed in an oven at 75 °C to dry them until a constant weight was achieved.

Determination of the Cd content: First, the dried plant samples were digested using the wet digestion method. Then, the Cd contents of the roots and shoots were measured using an atomic absorption spectrophotometer (AA-6300, SHIMADZU, Japan).

We used the translocation factor (TF) (Equation (1)) to evaluate the capacity of the selected plants to translocate Cd from their roots to their shoots.

TF = C_shoot_/C_root_, (1)

where C_shoot_ and C_root_ are the Cd concentrations (mg kg^−1^, dry weight) in the shoots and roots, respectively.

The amount of Cd accumulation was then calculated using Equation (2):

Cd accumulation = Cd_shoot (or root)_ × W_shoot (or root)_, (2)

where W_shoot (or root)_ represents the dry biomass (g, dry weight) of the shoot or root, respectively.

### 4.4. Statistical Analyses

The data were organized, examined, and graphed utilizing Excel 2021 and Origin 2022. An analysis of variance and least significant difference (LSD) test for treatment comparisons was conducted using SPSS 26.0 software. A Pearson correlation analysis was also performed using SPSS, incorporating the data of PEG-6000 concentrations, root morphology, Cd content, and Cd accumulation. A path analysis of the association between PEG-6000 treatment and root morphology, Cd concentration, and accumulation was conducted using SPSSPRO (http://www.spsspro.com, accessed on 22 August 2023).

## 5. Conclusions

The present study aimed to examine the impact of drought stress on seedling biomass, morphological development, and Cd uptake and accumulation in two varieties of low- and high-grain Cd-accumulating species under specific Cd pollution backgrounds. To achieve this, a hydroponic experiment was conducted to create a uniform Cd-polluted environment and simulate various drought gradient treatments with PEG-6000. The analysis revealed that the interaction between drought treatment and Cd stress resulted in a decrease in seedling biomass and an increase in the root–crown ratio. The root morphology (e.g., total root length, surface area, and volume) of XM and YM was suppressed, and the content and accumulation of Cd in roots and shoots showed a decreasing trend. Furthermore, the trends of both root morphology and Cd uptake and accumulation were found to be enhanced with increasing concentration of PEG-6000 treatment. Correlation analysis showed a significant negative correlation between PEG-6000 concentration and root length, surface area, and volume, which was one of the main mechanisms for reducing Cd uptake and accumulation in XM and YM. By considering the outcomes of the correlation analysis alongside the changes in biomass and morphology of seedlings, we could conclude that drought stress without significant reduction of biomass can be used to reduce the uptake and accumulation of Cd in wheat plants. Path analysis further indicated that changes in root morphology (root length) and a decrease in Cd concentration in roots might be the main direct pathways for achieving the aforementioned effects under drought stress. Furthermore, the inhibitory effects appeared to be consistent across different genotypes. The results of this study will contribute to the establishment of a theoretical foundation and serve as a valuable reference for the research on water management in Cd-contaminated farmland.

## Figures and Tables

**Table 1:** Effects of different drought stress induced by polyethylene glycol (PEG-6000) treatments on the biomasses and root/shoot ratios of XM (high-grain Cadmium (Cd)-accumulating genotype) and YM (low-grain Cd-accumulating genotype) under 100 µmol L^−1^ of Cd stress.

Treatment	Dry Biomass (g)			Root/Shoot Ratio
	Roots	Shoots	Whole Plant	
Cd-only XM	0.070 ± 0.02 a	0.263 ± 0.04 a	0.332 ± 0.12 a	0.274 ± 0.03 d
Cd-only YM	0.057 ± 0.01 a	0.212 ± 0.03 ab	0.269 ± 0.04 abc	0.270 ± 0.02 d
Cd + 5% PEG-6000-XM	0.064 ± 0.00 a	0.196 ± 0.01 ab	0.260 ± 0.01 abc	0.328 ± 0.03 cd
Cd + 5% PEG-6000-YM	0.068 ± 0.01 a	0.211 ± 0.05 ab	0.279 ± 0.06 ab	0.326 ± 0.02 cd
Cd + 10% PEG-6000-XM	0.061 ± 0.00 a	0.150 ± 0.02 bc	0.210 ± 0.02 bc	0.410 ± 0.06 c
Cd + 10% PEG-6000-YM	0.053 ± 0.00 a	0.146 ± 0.02 bc	0.199 ± 0.02 bc	0.369 ± 0.04 c
Cd + 15% PEG-6000-XM	0.069 ± 0.01 a	0.107 ± 0.01 c	0.176 ± 0.02 c	0.652 ± 0.10 a
Cd + 15% PEG-6000-YM	0.060 ± 0.00 a	0.111 ± 0.01 c	0.171 ± 0.01 c	0.539 ± 0.02 b
Significance based on two-way ANOVA (*p*-value)				
Varieties	0.118	0.314	0.232	0.077
Stress Level	0.381	0.000	0.000	0.000
Varieties * Stress Level	0.442	0.057	0.100	0.208

Data are presented as the mean ± SD (*n* = 3). The different letters indicate significant differences at *p* < 0.05 according to a least significant difference (LSD) test. * means the interaction between Varieties and Stress level.

**Table 2:** Effects of different drought stress induced by polyethylene glycol (PEG-6000) treatments on the Cadmium (Cd) concentrations and transfer coefficients of XM (high-grain Cd-accumulating genotype) and YM (low-grain Cd-accumulating genotype) under 100 µmol L^−1^ of Cd stress.

Treatment	Cd Concentration (mg kg^−1^)		Translocation Factor
	Roots	Shoots	
Cd-only XM	182.137 ± 3.038 a	94.485 ± 4.129 b	0.519
Cd-only YM	186.217 ± 3.482 a	105.840 ± 4.492 a	0.568
Cd + 5% PEG-6000-XM	140.981 ± 8.167 c	46.808 ± 2.823 d	0.332
Cd + 5% PEG-6000-YM	162.916 ± 3.535 b	45.647 ± 0.487 d	0.280
Cd + 10% PEG-6000-XM	86.234 ± 2.760 e	35.343 ± 4.040 e	0.410
Cd + 10% PEG-6000-YM	97.201 ± 1.104 d	55.173 ± 1.081 c	0.568
Cd + 15% PEG-6000-XM	67.679 ± 1.945 f	20.123 ± 1.852 f	0.297
Cd + 15% PEG-6000-YM	61.636 ± 4.083 f	20.565 ± 0.513 f	0.334
Significance based on two-way ANOVA (*p*-value)			
Varieties	0.000	0.000	0.000
Stress Level	0.000	0.000	0.000
Varieties * Stress Level	0.000	0.000	0.000

The different letters indicate significant differences at *p* < 0.05 according to a least significant difference (LSD) test. * means the interaction between Varieties and Stress level.

**Table 3:** Effects of different drought stress induced by polyethylene glycol (PEG-6000) treatments on Cadmium (Cd) accumulation in XM (high-grain Cd-accumulating genotype) and YM (low-grain Cd-accumulating genotype) under 100 µmol L^−1^ of Cd stress.

Treatment	Cd Accumulation (µg)		
	Roots	Shoots	Whole Plants
Cd-only XM	12.713 ± 1.893 a	24.880 ± 3.596 a	37.593 ± 5.053 a
Cd-only YM	10.621 ± 1.280 ab	22.383 ± 3.180 a	33.004 ± 4.387 a
Cd + 5% PEG-6000-XM	9.009 ± 0.371 b	9.176 ± 1.018 b	18.184 ± 0.836 b
Cd + 5% PEG-6000-YM	11.136 ± 2.257 ab	9.637 ± 0.874 b	20.773 ± 2.947 b
Cd + 10% PEG-6000-XM	5.222 ± 0.326 c	5.233 ± 0.431 cd	10.455 ± 0.306 cd
Cd + 10% PEG-6000-YM	5.194 ± 0.508 c	8.029 ± 1.002 bc	13.223 ± 1.385 c
Cd + 15% PEG-6000-XM	4.689 ± 0.905 c	2.158 ± 0.442 d	6.847 ± 1.136 d
Cd + 15% PEG-6000-YM	3.705 ± 0.475 c	2.289 ± 0.195 d	5.994 ± 0.656 d
Significance based on two-way ANOVA (*p*-value)			
Varieties	0.630	0.768	0.984
Stress Level	0.000	0.000	0.000
Varieties * Stress Level	0.049	0.134	0.097

The different letters indicate significant differences at *p* < 0.05 according to a least significant difference (LSD) test. * means the interaction between Varieties and Stress level.

**Table 4:** Effects of different drought stress induced by polyethylene glycol (PEG-6000) treatments on the parameters for fine (0–1.0 mm) and thick (>1 mm) roots of XM (high-grain Cadmium (Cd)-accumulating genotype) and YM (low-grain Cd-accumulating genotype) under 100 µmol L^−1^ of Cd stress.

Treatment	Root Length (cm)		Root Surface Area (cm^2^)		Root Volume (cm^3^)	
	0–1.0 mm	>1.0 mm	0–1.0 mm	>1.0 mm	0–1.0 mm	>1.0 mm
Cd-only XM	1412.825 ± 51.919 a	1.071 ± 0.130 a	85.606 ± 7.268 a	0.355 ± 0.019 b	0.658 ± 0.061 bc	0.010 ± 0.000 b
Cd-only YM	1082.320 ± 44.730 c	0.331 ± 0.029 e	74.998 ± 9.993 abc	0.125 ± 0.005 d	0.566 ± 0.077 d	0.003 ± 0.000 d
Cd + 5% PEG-6000-XM	1221.233 ± 17.085 b	1.221 ± 0.158 a	85.458 ± 1.992 a	0.420 ± 0.035 a	0.693 ± 0.018 ab	0.012 ± 0.002 a
Cd + 5% PEG-6000-YM	1190.430 ± 68.234 b	0.896 ± 0.093 b	84.309 ± 15.621 ab	0.337 ± 0.002 b	0.742 ± 0.026 a	0.010 ± 0.001 b
Cd + 10% PEG-6000-XM	1179.531 ± 30.926 b	0.530 ± 0.053 cd	78.705 ± 0.187 abc	0.148 ± 0.011 d	0.603 ± 0.005 cd	0.005 ± 0.001 c
Cd + 10% PEG-6000-YM	1076.758 ± 65.526 c	0.379 ± 0.045 de	67.237 ± 4.917 bc	0.133 ± 0.011 d	0.469 ± 0.044 e	0.003 ± 0.000 d
Cd + 15% PEG-6000-XM	1145.318 ± 43.325 bc	0.546 ± 0.077 c	80.233 ± 14.556 abc	0.193 ± 0.009 c	0.563 ± 0.021 d	0.006 ± 0.001 c
Cd + 15% PEG-6000-YM	982.864 ± 35.210 d	0.037 ± 0.003 f	64.543 ± 2.559 c	0.012 ± 0.001 e	0.467 ± 0.035 e	0.000 ± 0.000 e
Significance based on two-way ANOVA (*p*-value)						
Varieties	0.000	0.000	0.017	0.000	0.001	0.000
Stress Level	0.000	0.000	0.080	0.000	0.000	0.000
Varieties * Stress Level	0.000	0.000	0.564	0.000	0.009	0.000

The different letters indicate significant differences at *p* < 0.05 according to a least significant difference (LSD) test. * means the interaction between Varieties and Stress level.

## Data Availability

Data will be made available on request.
